# Colloidal Self-Assembled Patterns Maintain the Pluripotency and Promote the Hemopoietic Potential of Human Embryonic Stem Cells

**DOI:** 10.3389/fcell.2021.771773

**Published:** 2021-11-16

**Authors:** Jiao Lin, Jiahui Zeng, Wencui Sun, Kun Liu, Myagmartsend Enkhbat, Danying Yi, Javad Harati, Jiaxin Liu, Peter Kingshott, Bo Chen, Feng Ma, Peng-Yuan Wang

**Affiliations:** ^1^ Shenzhen Key Laboratory of Biomimetic Materials and Cellular Immunomodulation, Shenzhen Institute of Advanced Technology, Chinese Academy of Sciences, Shenzhen, China; ^2^ Stem Cell Center, Institute of Blood Transfusion, Chinese Academy of Medical Sciences and Peking Union Medical College (CAMS and PUMC), Chengdu, China; ^3^ Department of Chemistry and Biotechnology, Swinburne University of Technology, Hawthorn, VIC, Australia

**Keywords:** colloidal self-assembly, artificial ECM, pluripotent stem cells, hematopoiesis, blood cells, focal adhesion

## Abstract

The generation of blood cells in a significant amount for clinical uses is still challenging. Human pluripotent stem cells-derived hemopoietic cells (hPSC-HCs) are a promising cell source to generate blood cells. Previously, it has been shown that the attached substrates are crucial in the maintenance or differentiation of hPSCs. In this study, a new family of artificial extracellular matrix (ECM) called colloidal self-assembled patterns (cSAPs: #1–#5) was used for the expansion of mouse and human PSCs. The optimized cSAP (i.e., #4 and #5) was selected for subsequent hemopoietic differentiation of human embryonic stem cells (hESCs). Results showed that the hematopoietic potential of hESCs was enhanced approx 3–4 folds on cSAP #5 compared to the flat control. The cell population of hematopoietic progenitors (i.e., CD34^+^CD43^+^ cells) and erythroid progenitors (i.e., CD71+GPA+ cells) were enhanced 4 folds at day 8 and 3 folds at day 14. RNA sequencing analysis of cSAP-derived hESCs showed that there were 300 genes up-regulated and 627 genes down-regulated compared to the flat control. The enriched signaling pathways, including up-regulation (i.e., Toll-like receptor, HIF-1a, and Notch) or down-regulation (i.e., FAs, MAPK, JAK/STAT, and TGF-β) were classic in the maintenance of hESC phenotype Real time PCR confirmed that the expression of focal adhesion (PTK2, VCL, and CXCL14) and MAPK signaling (CAV1) related genes was down-regulated 2-3 folds compared to the flat control. Altogether, cSAP enhances the pluripotency and the hematopoietic potential of hESCs that subsequently generates more blood-like cells. This study reveals the potential of cSAPs on the expansion and early-stage blood cell lineage differentiation of hPSCs.

## 1 Introduction

Substrate for anchor-dependent cells is crucial for self-renew and lineage commitment, including human embryonic stem cells (hESCs) (Murphy et al., 2014). In the last decades, substrates with different nanostructures such as nanogrooves and nanopillars have been applied to manipulate cell fate. Nanostructured surfaces can be further modified chemically using coatings or grafting technology to enhance bio-functionality. However, these surfaces are still far from an extracellular matrix (ECM) like substrate. ECM mimetic surface presenting hierarchical structures and multiple chemistries are rare to be found (Wang et al., 2016a).

Recently, a new family of substrates composed of various colloidal particles with different sizes and materials named self-assembled patterns (cSAPs) was developed in our group (Wang et al., 2015a). The particles can be pre- or post-modified, ultimately providing a complex surface of the cSAPs (Diba et al., 2019). Surface topography, roughness, hydrophilicity, chemistry, and even stiffness can be fine-tuned. The behaviors of human stem cells and adult cells have been investigated on the cSAPs (Wang et al., 2016b; Cui et al., 2019). Cell reprogramming of human fibroblasts into human induced pluripotent stem cells (hiPSCs) has also been studied on these new substrates (Wang et al., 2016c). cSAPs have shown the potential to control cell adhesion and subsequently the fate decision of cells.

Generation of blood cells *in vitro* with a significant amount for clinical uses is still challenging. Human pluripotent stem cells (hPSCs), including hESCs and hiPSCs, are promising sources to generate blood-like cells. The hematopoietic potential of hPSCs has a significant application in the cure of blood-related diseases such as Thalassemia (Papapetrou, 2017) or hemophilia (Wang et al., 2013). Several co-culture differentiation systems, such as the OP9 stromal cells and the aorta–gonad–mesonephros-derived stromal cells (AGM-S3) (Ledran et al., 2008), have been established for blood-like cell generation. These systems use a nature-inspired microenvironment to stimulate definitive hematopoiesis *in vitro*. These systems are useful to identify the function of critical genes in normal or abnormal hematopoiesis. For example, the AGM-S3 co-culture system could be used to check the detailed cellular and molecular mechanism of hematopoiesis influenced by the critical gene (Chen et al., 2017; Zeng et al., 2020). It also has potential utility in screening for compounds that promote human hematopoiesis, which is possible to set up a high throughput screening system for compound function screening using the AGM-S3 co-culture system (Chang et al., 2019). However, the current approach *in vitro* blood cell generation is far from satisfying clinical needs in terms of quality and number (Moreno-Gimeno et al., 2010). Therefore, the improvement of the efficiency of hematopoietic differentiation of hPSC-HCs is essential.

In this proof-of-concept study, human and mouse PSCs’ morphology and growth were screened on five cSAPs. The selected cSAPs were used to expand and stimulate hESCs used for the downstream hematopoietic differentiation into HCs and blood-like cells. The outcomes imply that the expansion of hESCs is a crucial step prior to the hematopoietic differentiation using the stromal cell co-culture system, and the cSAP is a valuable tool for *in vitro* blood cell production.

## 2 Materials and Methods

### 2.1 Colloidal Self-Assembled Patterns

**FIGURE 1:**
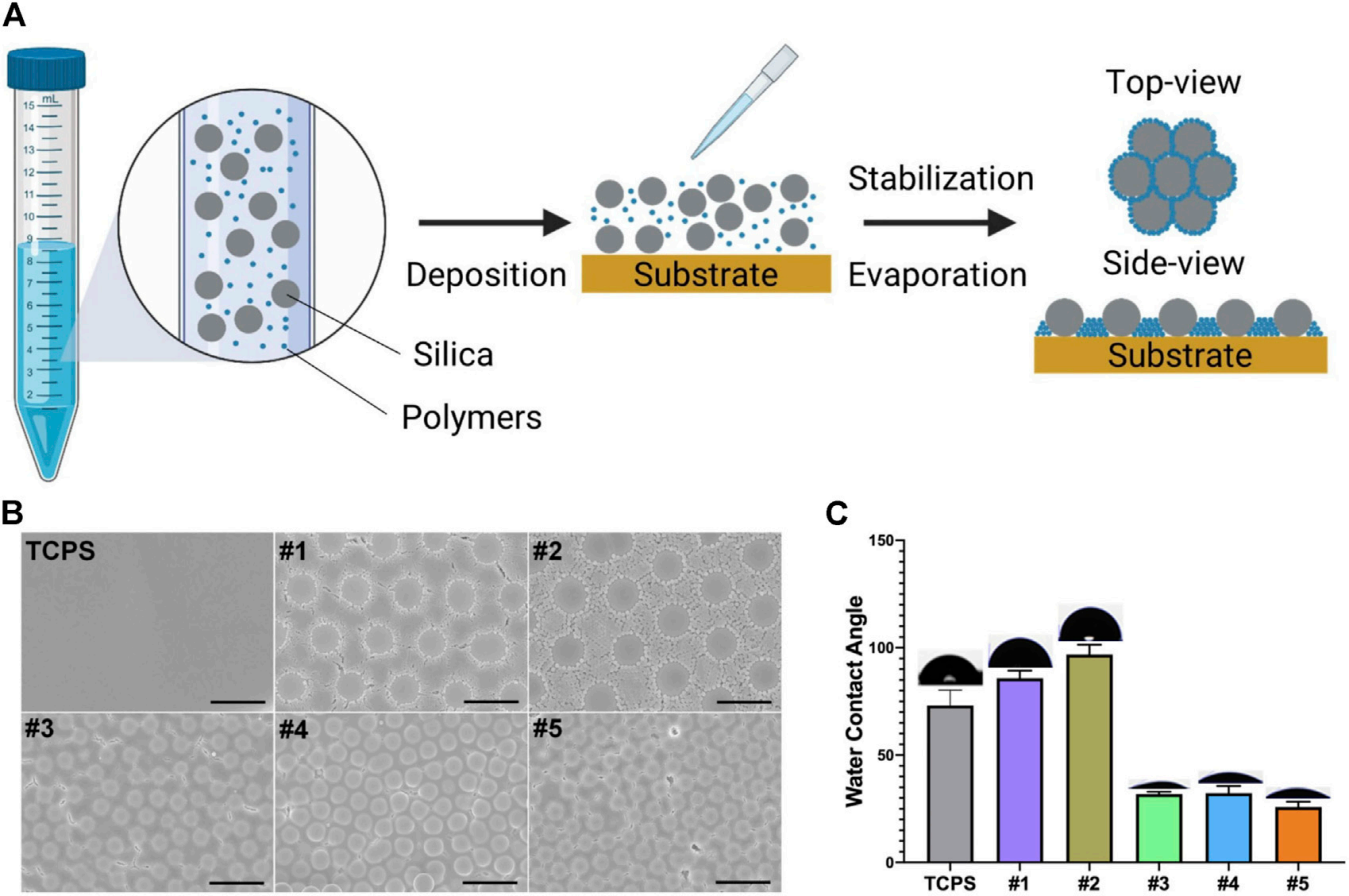
Fabrication and characterization of cSAPSs. **(A)** Scheme of cSAPs preparation. **(B)** SEM of TCPS and five selected cSAPs. Scale bar represents 5 μm. **(C)** Surface wettability of the substrates was analyzed using water contact angle test (degrees). Five spots on each surface were analyzed (*n* = 5). Error bar = STDEV.

Five substrates were selected from our library and fabricated according to our previous protocol (Wang et al., 2016b) (Figure 1A). Briefly, cSAPs #1 and #2 was composed of SiO_2_ with 5 μm diameter and polystyrene (PS) with 200 or 400 nm diameter, #3, #4, and #5 was composed of SiO_2_ with 2 μm diameter and PS with 65 nm diameter, carboxy-PS (PSC) with 50 or 100 nm diameter, and the cSAPs were fabricated within the 24-well tissue culture plates (TCPS, Falcon). TCPS was the flat control group in this study.

Scanning electron microscopy (SEM, ZEISS SUPRA ® 55, Carl Zeiss, Germany) and water contact angle (WCA) was used to characterize the surface structures and wettability of these cSAPs using an automated contact angle measurement device (PT-705B, Precise Test, China) at room temperature. Other detailed characterizations have been done previously (Wang et al., 2016b).

### 2.2 Cell Culture

H1 hESCs and human induced pluripotent stem cells (hiPSCs) were routinely cultured in Matrigel (BD, United State) coated 60 mm Petri dishes with mTeSR1 medium (Stem Cells, United State), and passaged using ReleSR (Stem Cells, United State) with a ratio of 1:3 every other day. E14 mESCs and mouse induced pluripotent stem cells (miPSCs) were routinely maintained on gelatin (Yeasen, China) coated 6-well tissue culture plates in mouse ESC medium (DMEM-knockout, GIBCO, United State) containing 20% FBS, 1% NEAA, 1% l-Glutamine, 1% penicillin, and streptomycin (P/S, GIBCO, United State), 0.15 mM 1-thioglycerol (Sigma, United State), 1000 U/mL leukemia inhibitory factor (Millipore), and two chemical inhibitors (2i), 3 μM CHIR99021 and 1 μM PD0325901 (Stemgent, United State). mESCs and miPSCs were passaged using trypsin-EDTA (Invitrogen, United State) with a ratio of 1:6 every other day. All the medium was changed daily.

All cells used in this study were passaged three times on cSAPs prepared in 24-well tissue culture plate or control surfaces coated by Matrigel or Gelatin for further research. The experimental group was named cSAP #X (X = 1, 2, 3, 4, 5) and TCPS (as a flat control). Cell inoculatiion density of mESCs and miPSCs in experimental group was 2  ×  10^4^ cells/cm^2^. Cell density of hESCs and hiPSCs was adjusted according to the colony quantity. Cell culture in experimental group was same to the routinely cultured cells, mESCs and miPSCs was passaged at a ratio of 1:6, hESCs and hiPSCs was passaged at a ratio of 1:3. Medium was changed every day. Cell morphology was captured every day by an inverted microscope (Olympus-IX71).

### 2.3 Real-Time qPCR

Total RNA was extracted from cells with the MiniBEST universal RAN extraction Kit (Takara, Japan). cDNA was made with 1 mg of total RNA based on the manufacturer’s protocol using the PrimeScript^TM^ RT Master Mix (Takara, Japan), and subsequent real-time qPCR was carried out in triplicates, using the 2 × RealStar Green Mixture (GENE STAR, China) on Light Cycler 96 System cording to manufacturer’s instructions (Roche, United State). Amplifications were performed using the following conditions: 95°Cfor 2 min, followed by 40 cycles of 95°C for 15 s, 60°C for 15 s, and 72°C for 30 s. Gene expression was normalized to *Gapdh*, and then the TCPS control. The sequences of all primers used were listed in Supplementary Table S1.

### 2.4 Hemopoietic Differentiation of Human Embryonic Stem Cells

hESCs were co-cultured with mouse stromal cell line AGM-S3 to induce their differentiation into hematopoietic cells, as reported before (Chen et al., 2017). hESCs were cut into small squares of 1.5 × 10^3^ cells, and then plated onto the irradiated AGM-S3 cells and cultured in the hPSC maintenance medium [high glucose Dulbecco’s modified Eagle’s medium, DMEM, F-12 nutrient mixture, 20% knockout serum replacement (KSR), 1% l-glutamine, 1% non-essential amino acid solution (NEAA; Gibco), and 5 ng/ml basic FGF (b-FGF; Wako)] for 3 days (Chen et al., 2017). The medium was changed to the hematopoiesis-inducing medium (Iscove’s Modified Dulbecco’s Medium (IMDM) containing 10% fetal bovine serum (FBS; Hyclone), 1% NEAA (Gibco), 60 ng/ml ascorbic acid (Sigma), and 20 ng/ml vascular endothelial growth factor (VEGF; Wako)), referred as day 0 (D0) (Chen et al., 2017; Zhou et al., 2019). The culture medium was replaced every day. The co-cultures were dissociated by 0.05–0.25% trypsin-EDTA (Invitrogen) at D8 or D14 for further analysis. This experiment was run at least three times in parallel.

### 2.5 Flow Cytometry

Flow cytometry experiments were performed according to a previous report (Chen et al., 2017). Briefly, the co-cultured cells at D8 or D14 were dissociated with 0.25% trypsin-EDTA solution. The cell suspension was obtained by filtration through a 70-μm nylon mesh. Before immunostaining, cells were blocked by rabbit serum at 4°C for 30 min. Cells were stained with the anti-CD34/CD43 antibodies (at D8 and D14), and anti-CD34/CD45 or anti-GPA/CD71 antibodies (at D14) at 4°C for 30 min (Supplementary Table S2). Flow cytometric analysis was performed using the FACS Canto II system (BD Biosciences). All FACS data were analyzed using FlowJo 10 software.

### 2.6 Hematopoietic Colony-forming Assay

According to the previous studies, the hematopoietic colony-forming assay of co-cultured cells was performed (Chen et al., 2017; Sun et al., 2020). Briefly, co-cultured cells were dissociated by 0.25% trypsin/EDTA into single cells at day 14.5 × 10^4^ cells were suspended in 80% MethoCult H4230 (Stem cells) containing 100 ng/ml stem cell factor (SCF), 100 ng/ml interleukin-6 (IL-6), 10 ng/ml interleukin-3 (IL-3), 10 ng/ml Fms-related tyrosine kinase three ligand (FL), 10 ng/ml thrombopoietin (TPO), 10 ng/ml granulocyte-macrophage colony-stimulating factor (GM-CSF), and 4 units/ml erythropoietin (EPO), and then incubated in 5% CO_2_ at 37°C for another 14 days. The number of colony-forming unit erythroid (CFU-E) colonies was counted at 7–10 days, while the number of burst-forming unit-erythroid (BFU-E), colony-forming unit-mixed (CFU-Mix), and colony-forming unit-granulocyte/macrophage (CFU-GM) colonies were counted at 12–14 days. This experiment was run at least three times in parallel.

### 2.7 RNA Sequencing

H1 hESCs cultured on cSAPs and flat control (TCPS) for two passages (4 days/passage) were dissociated with 0.5 mM EDTA for RNAseq. Total RNA of each 1 × 10^6^ cells was extracted by 1 ml TRIzol (Life Technologies) and purified according to the standard instruction. RNA Sequence analysis was performed by BGI Tech (Shenzhen, China). The analysis of gene function was performed with the multi-group data mining system of Dr. Tom (http://report.bgi.com). Gene changes on cSAP #5 compare to TCPS over 2-fold were defined as significant.

### 2.8 Immunofluorescence Staining

Cells were cultured in multi-well plates were fixed with 4% paraformaldehyde (PFA) for 30 min at 4°C, and washed three times with wash buffer (5% FBS in PBS). They were then incubated with membrane permeation reagent (PBS containing 0.3% Triton-100 and 5% FBS) at 4°C for 30 min, stained overnight at 4°C with anti-OCT4/SOX2/SSEA4 antibodies (mouse anti-human), washed three times with PBS containing 5% skim milk, and then incubated for 30 min at room temperature with secondary antibodies (FITC-conjugated secondary Ab, goat anti-mouse) (Supplementary Table S2). Nuclei were labeled with DAPI. After washing three times with PBS, the sample was imaged under a fluorescence microscope.

### 2.9 Statistical Analysis

All data are presented as means ± SD; statistical analyses were performed by GraphPad Prism 8 using the Student’s t-test, one-way ANOVA with Dunnett post hoc test, and two-way ANOVA with Tukey’s post hoc test. *p* < 0.05 was considered significant.

## 3 Results

### 3.1 Colloidal Self-Assembled Patterns Characterization

cSAPs were fabricated by mixing different particles together and depositing them on the tissue culture plates (TCPS); after evaporation, particles were distributed on the surface according to the principle of self-assembly (Figure 1A). The surface topography of cSAPs was measured by SEM and showed that the large particles and small particles were orderly distributed on the surfaces (Figure 1B). Due to the differences of the surface on chemestry and topography, surface wettability showed that water contact angle (WCA) of cSAP #1 and #2 (SiO_2_ = 5 μm; PS = 200 and 400 nm) was more hydrophobic (85.8 ± 3.5 and 96.8 ± 4.6 degree), than the cSAP #3, #4, and #5 (SiO2 = 2 μm; PS = 65 nm, PSC = 50 and 100 nm), and the WCA of cSAP #3, #4, and #5 was 31.8 ± 1.1, 32.3 ± 3.2, and 25.8 ± 2.4 degree, respectively (Figure 1C).

### 3.2 Colloidal Self-Assembled Patterns Maintain Pluripotency of miPSCs, hiPSCs, and hESCs

**FIGURE 2:**
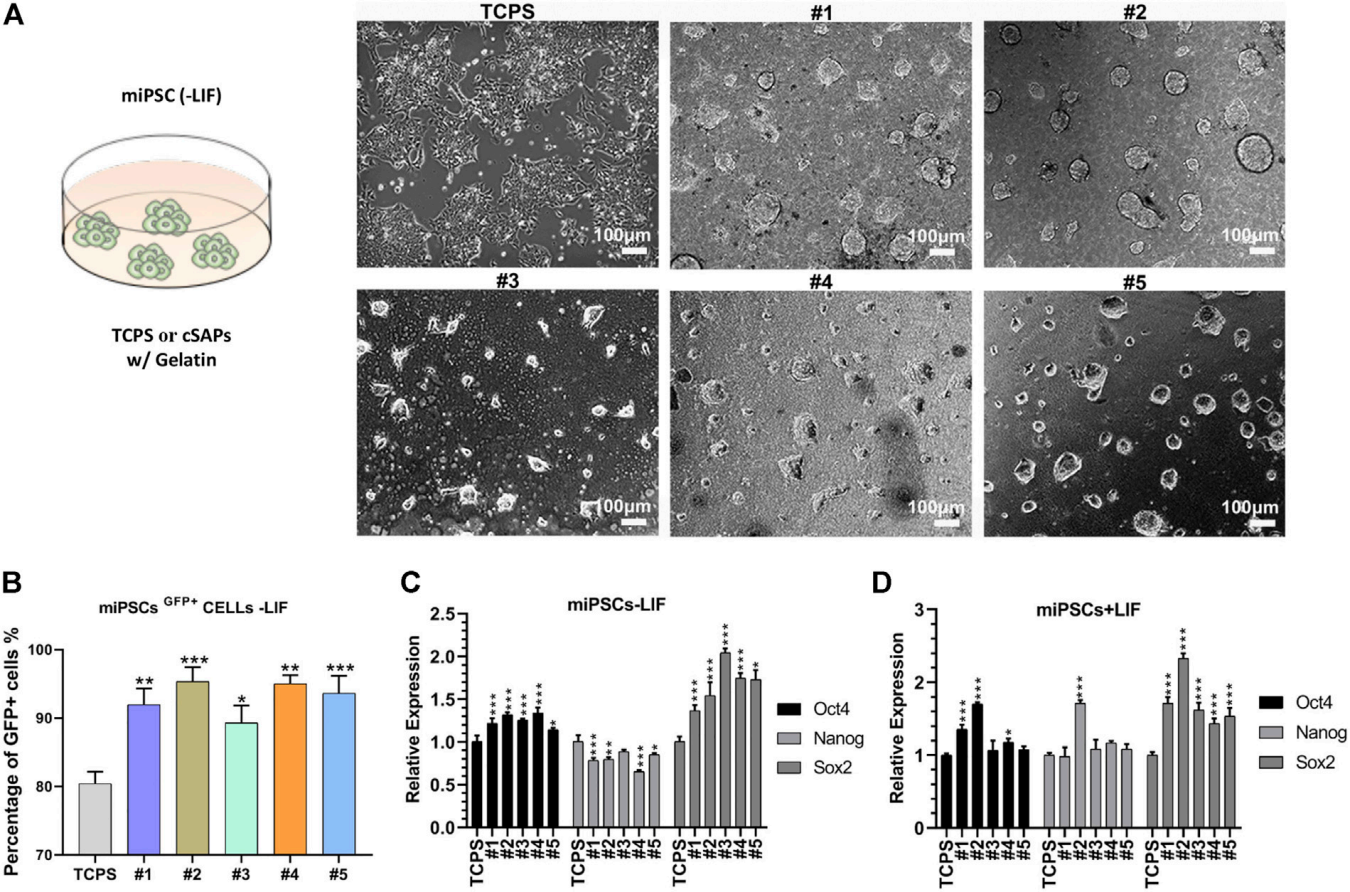
Pluripotency of miPSCs on different substrates coated with gelatin. **(A)** Colony morphology of miPSCs cultured on different surfaces after day 3. Scale bar = 100 μm. **(B)** FACS analysis of the Oct4+ cells without LIF. qPCR analysis of pluripotent markers, *Oct4, Nanog, and Sox2* **(C)** without and **(D)** with LIF. Samples were triplicates (*n* = 3). Error bar = STDEV.

The colony morphology of miPSCs on cSAPs was significantly different from that on the TCPS control (Figure 2A). miPSCs colonies on cSAPs were more 3D-like, especially on the cSAP #1 and #2. The PSC colonies were dome-like morphology on the cSAP #4 and #5, neither 3D spheroids nor 2D-like colonies. miPSCs cultured on cSAPs without LIF (Leukemia Inhibitory Factor) had a higher percentage of *Oct4*-GFP positive cells after 7 days than TCPS without and even with LIF, indicating that cSAPs could maintain the pluripotency of PSCs (Figure 2B and Supplementary Figure S1A). Percentage of *Oct4*-GFP positive cells was more than 90% on cSAPs, except the cSAP #3, which was similar to the cells cultured within LIF (i.e., ∼80%).

Gene expression analysis showed that pluripotent markers (i.e., *Oct4, Nanog,* and *Sox2*) of miPSCs were similar (i.e., *Oct4* and *Nanog,* fold changes < 1.5) or higher (i.e., *Sox2,* fold changes > 1.5) on the cSAPs compared to the TCPS control without LIF (Figure 2C). Gene expression of miPSCs was also analyzed under LIF conditions. The results showed that *Sox2* expression was significantly higher on cSAPs than TCPS, while *Oct4* and *Nanog* were similar between surfaces (Figure 2D). The high expression of Sox2 may indicate neural stem and progenitor cells in some cSAPs groups (Ellis et al., 2004). The effects of cSAPs on miPSCs and mESCs were different because three genes of mESCs expressed similarity between surfaces (fold changes < 1.5, Figure 2C and Supplementary Figure S1B).

Gene expression of mesoderm markers of miPSCs was higher on cSAPs than the TCPS control without LIF (Supplementary Figure S1C). On average, the expression of *mT*, *mSnail2*, and m*Foxa2* on cSAPs, except cSAP #5, was significantly higher than the TCPS control (fold changes > 2, Supplementary Figure S1C).

**FIGURE 3:**
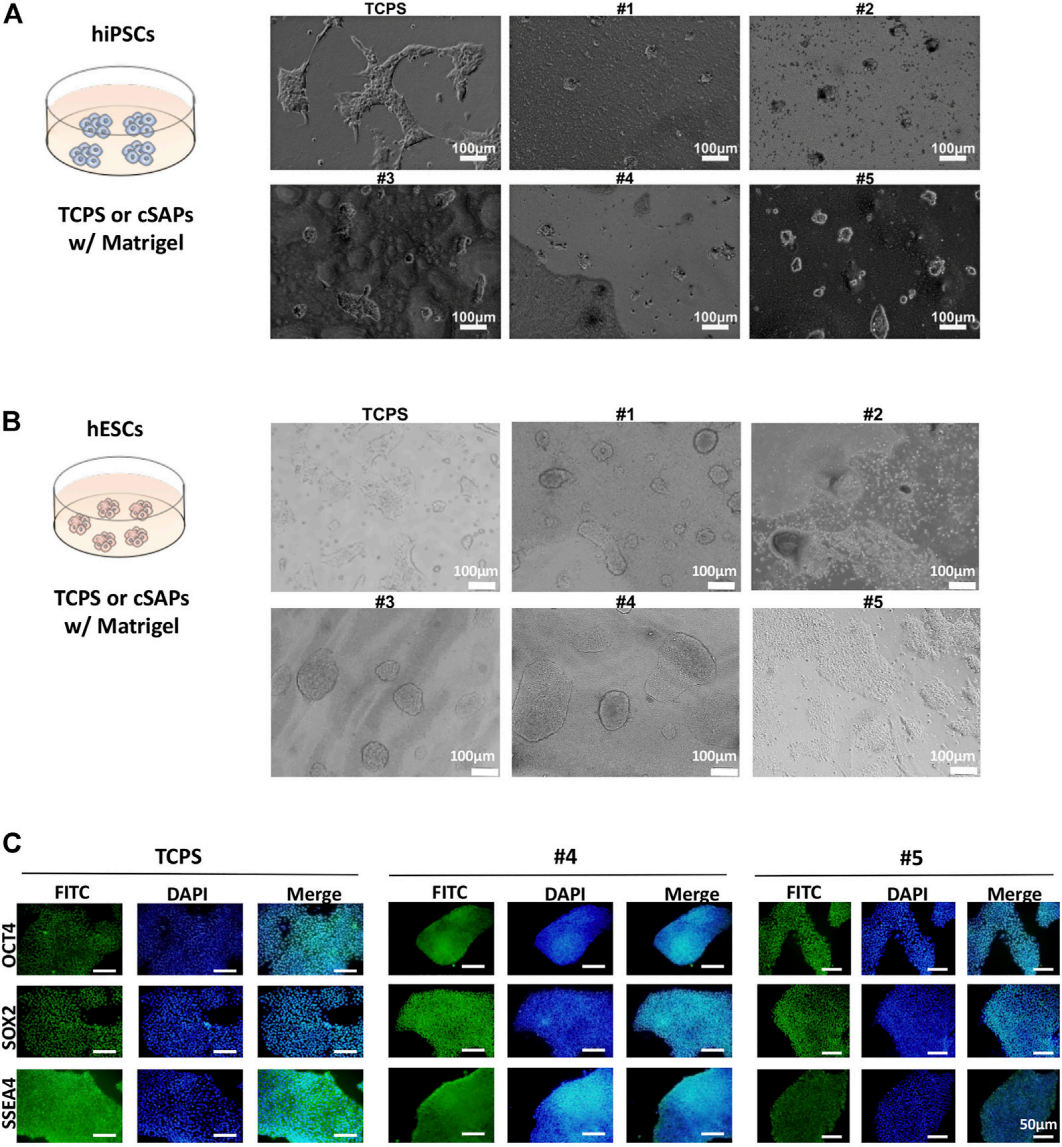
Pluripotency of hiPSCs and hESCs on different substrates coated with Matrigel. Colony morphology of **(A)** hiPSCs after 3 days and **(B)** hESCs after three passages cultured on different surfaces. Scale bar = 100 μm. **(C)** Immunostaining of pluripotent markers (OCT4, SOX2, and SSEA4) of hESCs after three passages’ culture on cSAPs and TCPS, Scale bars = 50 μm.

**FIGURE 4:**
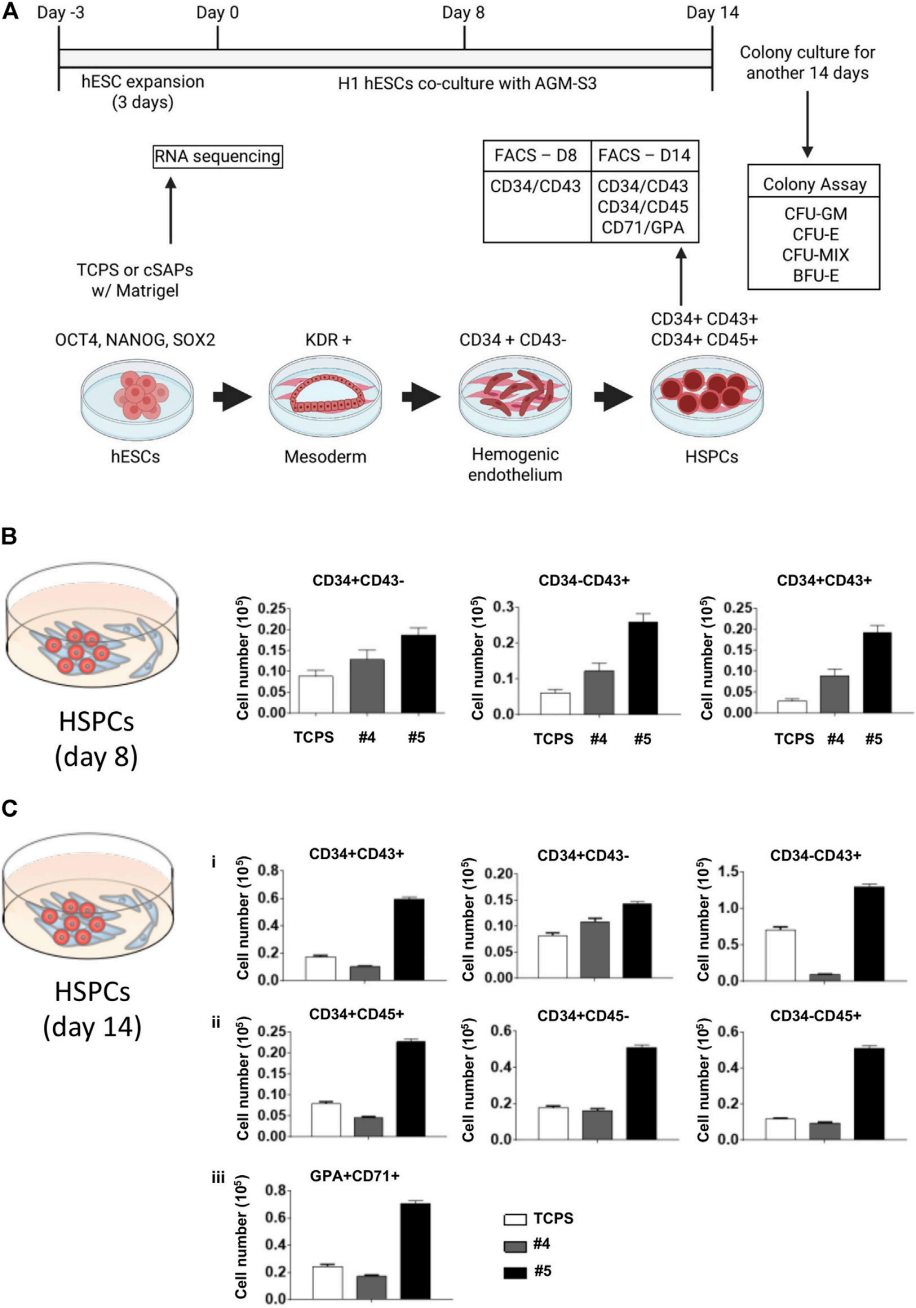
Hemopoietic differentiation of cSAP-cultured hESCs. **(A)** Scheme of the H1 hESC expansion and hemopoietic differentiation. FACS analysis of the hemopoietic efficiency of cSAP-cultured hESCs after **(B)** 8 days and **(C)** 14 days. Error bar = SEM.

**FIGURE 5:**
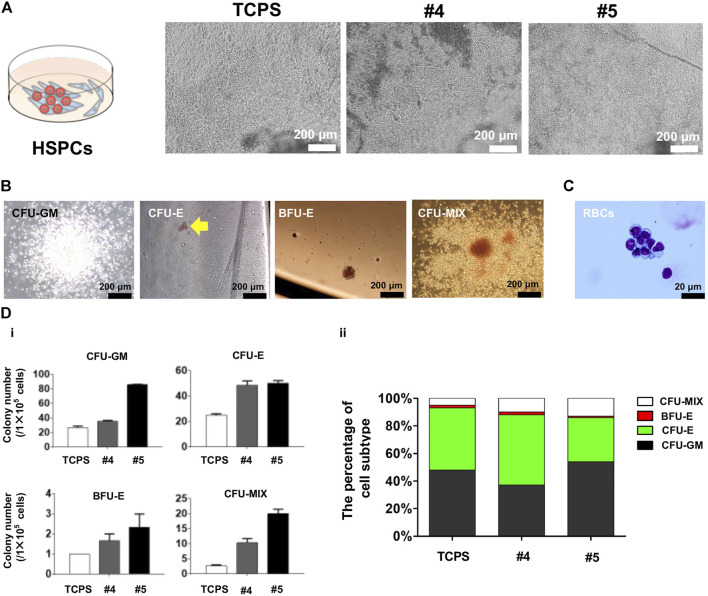
Characterization of blood-like cells derived from cSAP-cultured hESCs. **(A)** Cell morphology of hESC-HCs during co-culture with stromal AMG3 cells; **(B)** Colony culture assay after hESCs were cultured on cSAPs or TCPS in standard feeder-free culture method; **(C)** May-Grunwald-Giemsa (MGG) staining of erythroid cells differentiated from cSAP-derived hESCs; **(D)** The statistical analysis of (i) colony numbers of CFU-GM, CFU-E, BFU-E, or CFU-MIX colonies, and (ii) the percentage of colony subtype.

hiPSCs and hESCs also showed different colony morphology compared with TCPS and slight differences between cSAPs, similar to miPSCs on cSAPs showing a more 3D-like colony morphology (Figures 3A,B). For hESCs can not grow well without Matrigel, all surfaces used in culturing hESCs were precoated with Matrigel. FACS analysis (Figure 4 and Supplementary Figure S2) and the hematopoietic colony form assay (Figure 5) showed that only H1 hESCs cultured on cSAP#4 and #5 had better hematopoietic differentiation efficiency than the TCPS control when co-cultured with AGM-S3, so only #4 and #5 were chosen for further analysis of hESCs pluripothency. Besides, immunostaining of pluripotent markers, i.e., *OCT4, SOX2,* and *SSEA4,* showed that passaged hESCs were in high pluripotency on cSAPs, i.e., cSAP #4 and cSAP #5, and TCPS (Figure 3C).

### 3.3 Colloidal Self-Assembled Patterns Promote the Hematopoietic Potential of Human Embryonic Stem Cells

Cell number of hESC-HCs from cSAP-derived hESCs was higher than the TCPS control after 8 and 14 days (Figure 4 and Supplementary Figure S2). cSAPs, especially cSAP #5, strongly increased the cell populations of CD34^+^CD43^−^ (∼2-folds), CD34^−^CD43^+^ (∼4-folds), and CD34^+^CD43^+^ cells (∼5-folds; hematopoietic progenitors) at D8 (Figure 4B and Supplementary Figure S2A). Also, cell populations of CD34^+^CD43^−^ (∼1.5-folds), CD34^−^CD43^+^ (∼1.5-folds), and CD34^+^CD43^+^ (∼3-folds), CD34^+^CD45^−^ (∼2-folds), CD34^−^CD45^+^ (∼5-folds on cSAP #5), CD34^+^CD45^+^ (∼3-folds), and CD71 + GPA + cells (∼3-folds; erythroid progenitors) at D14 (Figure 4C and Supplementary Figure S2B).

The morphology of hESC-HCs on cSAPs was better compared to the TCPS control (Figure 5A). The colony assay proved that the hematopoiesis potentials of hESCs were significantly enhanced on cSAPs, especially #5. The numbers of CFU-GM, CFU-E, BFU-E, and CFU-MIX generated from cSAP-derived hESC-HCs were approx. 3-fold, 2-fold, 2-fold, and 7.5-fold higher than the TCPS control. Colony number represents the hematopoiesis potentials of H1 hESCs derived from different culture conditions to produce granulocyte/macrophage progenitors (CFU-GM), erythroid progenitors (CFU-E and BFU-E), and hematopoietic progenitors (CFU-MIX) after D14 of co-cultures (Figure 5B). The erythroid cells were confirmed by May-Grunwald-Giemsa (MGG) staining (Figure 5C). After co-culture differentiation and hematopoietic colony form culture, the absolute numbers of four kinds of colonies generated by H1 hESCs cultured on cSAPs#4 and #5 increased significantly compared with TCPS, especially cSAPs#5. Percentage of cell subtypes showed that H1 hESCs cultured on cSAPs# 5 were prone to generate the erythro-myeloid progenitor (EMP) in hematopoietic differentiation. Therefore, the cSAP #5-derived hESCs had significantly higher hematopoietic potentials to produce hematopoietic progenitors (i.e., CD34^+^CD45^+^ cells) and erythroid progenitors (i.e., GPA + CD71^+^ cells) compared to the TCPS control (Figure 5D **)**.

### 3.4 Colloidal Self-Assembled Patterns Alter the Transcriptome Profile of Cultured Human Embryonic Stem Cells

**FIGURE 6:**
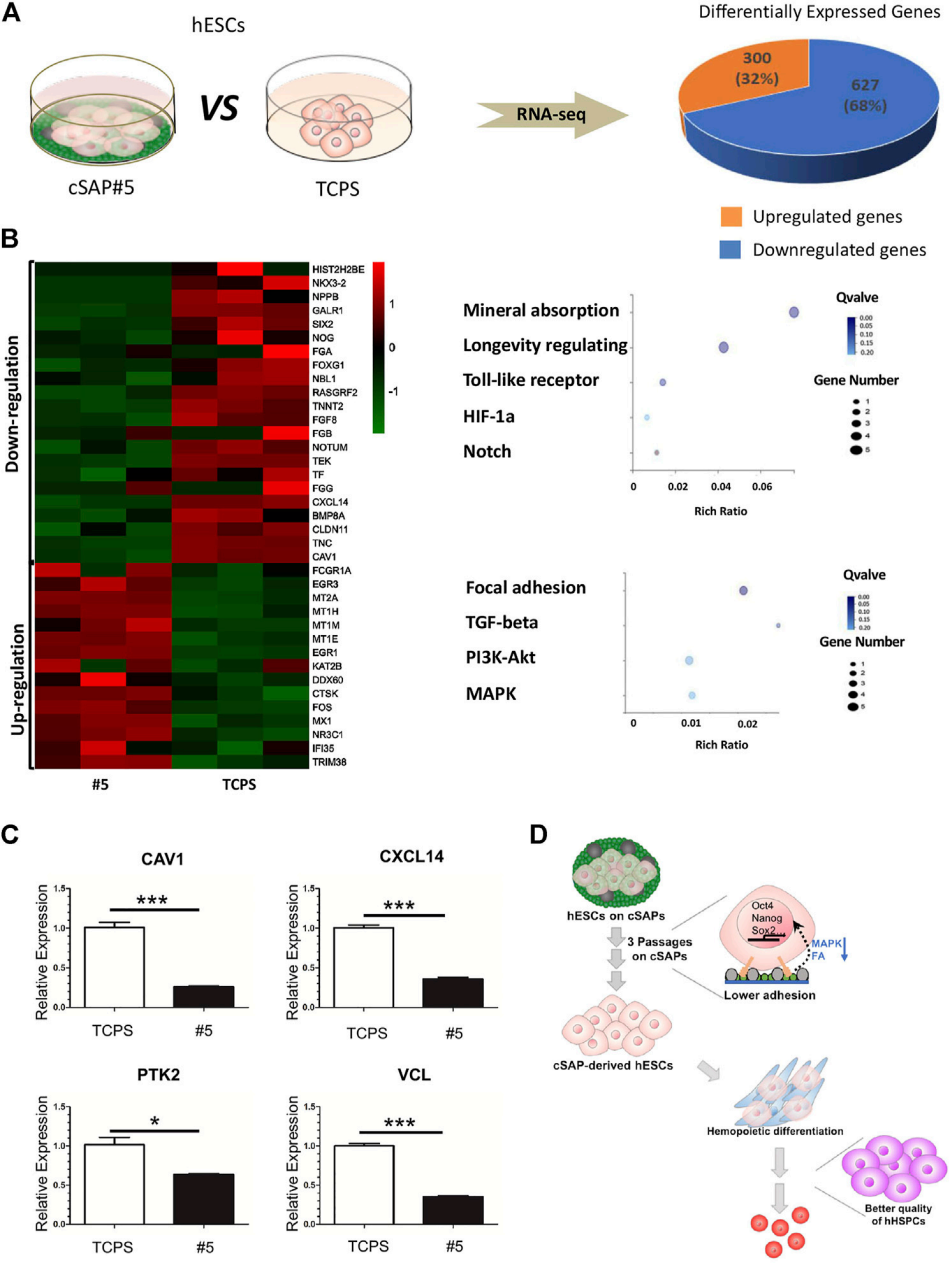
The transcriptional profile and qPCR analysis of cSAP-derived hESCs. **(A)** RNA-seq analysis of transcriptional profiling of cSAP #5-derived and TCPS-derived hESCs; **(B)** The differential expression genes (DEG) analysis. The selected genes were presented by heatmap, and the selected enriched pathways were shown by dot-plot. **(C)** qPCR results of CAV1, CXCL14, PTK2, and VCL expression after three passages’ culture (*n* = 4). **(D)** Schematic illustration of the role of cSAP in the maintenance of pluripotency and the improvement of the hemopoietic potential of hPSCs.

RNA-seq was used to analyze the change of cellular transcriptional profiling of hESCs after culturing on cSAP #5 (Figure 4A). Results showed that 300 genes (e.g., *EGR3, MT1M, EGR1, DDX60, FOS,* and *MX1*) were up-regulated significantly (32% of total differential expression genes), while the 627 genes (e.g., *NPPB, GALR1, SIX2, FOXG1, NBL1, RASGRF2, TNNT2, FGF8, NOTUM, TEK, TF, CXCL14, TNC,* and *CAV1*) were down-regulated significantly (68% of total differential expression genes) on cSAP #5 compared to the TCPS control (Figures 6A,B).

According to RNAseq analysis, the genes regulated on cSAPs were enriched in mineral absorption, longevity regulating, Toll-like receptor signaling, HIF-1a signaling, Notch signaling that were up-regulated, and the focal adhesion, TGF-β signaling, PI3K-Akt signaling, and MAPK signaling that were down-regulated (Figure 6B). According to the RNA-seq results we found that expression of CAV1 and CXCL14 was down-regulated (Figure 6B), and was consistent with qPCR verification (Figure 6C). CXCL14 is found concentrated in the focal adhesions, the down-regulated expression of CXCL14 (∼2.79 fold) on cSAP #5 resulted in lower expression of PTK2 (∼1.61 fold) and VCL (∼2.83 fold) (Figure 6C). The dot-plot also showed focal adhesion was enriched in down-regulated DEGs (Figure 6B). CAV1 can function as a scaffolding protein contribute to the activation of the MAP kinase pathway (Mineo et al., 1996; Engelman et al., 1998). In this study, the down-regulated MAPK pathway in Figure 6B may be the result of lower CAV1 (∼3.90 fold) expression on cSAP #5 (Figure 6C). Lower expression of CAV1 can promote hematopoietic differentiation of hESCs when co-cultured with stromal cells under hematopoietic induction condition (Choi et al., 2012). Therefore, cSAP-cultured hESCs having higher blood cell differentiation ability could be a consequence of down-regulation of focal adhesion and MAPK signaling (Figure 6D).

## 4 Discussion

Pluripotent stem cells (PSCs) can self-renew unlimitedly and differentiate into three germ layers *in vitro*. For example, under specific induction conditions, hPSCs can be differentiated sequentially to mesodermal cells, hematopoietic progenitor cells, and mature hematopoietic cells (Lim et al., 2013). *In vitro* hematopoietic differentiation facilitates a better understanding of hematopoiesis and embryonic development. Besides, the production of blood-like cells is significant in regenerative medicine.

Nowadays, three major *in vitro* differentiation systems have been established for the generation of hESC-derived HCs, including embryoid body (EB) culture, co-culture with feeder cells, and culture with extracellular matrix (ECM) protein-coated surfaces (Chen et al., 2014). Among these methods, the microenvironments for hESC expansion are critical to determining the subsequent HSC generation (Ma et al., 2008; Slukvin, 2013). ECM protein-coated surfaces, such as fibronectin, collagen IV, laminin, collagen I, entactin, and heparin-sulfate proteoglycan, have been developed to induce differentiation of mesodermal and hematopoietic lineages under more chemical defined conditions (Chen et al., 2011; Nakamura-Ishizu et al., 2012). However, few studies manipulated the adhesion status of hESCs prior to hematopoietic differentiation. Furthermore, previous studies heavily relied on biological modulators such as paracrine molecules and medium molecules, while the effects of biophysical stimulations during the process were rarely explored.

It has been demonstrated that the surface coating of cell culture substrate was able to modulate the fate of cells, including directional differentiation of mesenchymal and pluripotent stem cells (Wang et al., 2016a). Surface decoration with nanostructures and bioactive signals can reconstruct the stem cell niche’s microenvironments, which provide biophysical cues to the attached cells (Dalby et al., 2007). A previous study demonstrated that reduction of focal adhesions of mESCs was able to be maintained the cells in an undifferentiated and pluripotent state, while stronger cell adhesion resulted in stem cell differentiation (Taleahmad et al., 2017). The current study employed that colony morphology and adhesive force of PSCs varied on different cSAPs and the TCPS control. Further analysis demonstrated that PSCs’ pluripotent state was adhesion- and morphology-dependent, where more 3D-like colonies on cSAPs have higher pluripotency than the 2D culture. cSAPs can affect adhesion molecules and regulate the pluripotency of PSCs. This phenomenon is consistent with previous studies using different materials (Zujur et al., 2017).

Previous studies did show that physical cues on the substrate facilitate the generation of hESC-HCs. For example, graphene oxide (GO) had been reported to promote Endothelial-to-Hematopoietic Transition (EHT) and then the hESC-HC generation (Garcia-Alegria et al., 2016). In the current study, cSAPs don’t have superior conductivity or biofunctionality but hierarchical micro- and nanostructure and dual chemistries. It has been demonstrated that these properties will alter protein adsorption from the medium, cell adhesion, cell migration, and ECM synthesis of adhered cells (Wang et al., 2011; Wang et al., 2012; Wang et al., 2015b). cSAPs can maintain the pluripotent status of PSCs and stimulate mesodermal commitment. The chemical and physical properties of different cSAPs can maintain the pluripotency of PSCs to a different extent. According to a previous report, PSCs in a naïve state have more robust self-renew capability and limited potential toward lineage differentiation (Lee et al., 2017). Besides, the naïve PSCs need capacitation for triggering multi-lineage differentiation (Rostovskaya et al., 2019). According to the colony morphology, cSAPs may reverse the cells back into a naïve-like state (e.g., cSAP #2) or a state of capacitation (e.g., cSAP #5). The result implies that cSAP-derived hESCs have a better hematopoietic potential due to optimal cell adhesion and colony formation.

Controlling the cellular status of hESCs during expansion can enhance the hemopoietic potential in the AGM-S3 co-culture system. cSAPs coating with Matrigel or proteins can be seen as an artificial ECM that modulates the adhesion of hESCs during expansion. cSAP derived hESCs can generate 2–4 folds of HCs compared to the TCPS control. According to RNAseq data, a flock of genes related to mineral absorption, such metallothionein 1 (MT1) family, MX1, and NR3C1, and the signaling pathways of Toll-like receptor, HIF-1a, Notch were up-regulated. The genes related to cell adhesion and signaling pathways of MAPK, Jak-STAT, and TGF-β were down-regulated.

The changes of these genes and pathways play vital roles in hematopoiesis. For example, Notch signaling controls the hematopoiesis and inflammation process (Šućur et al., 2020). Toll-like receptors and HIF-1a signaling can influence the generation of hematopoietic stem and progenitor cells (Capitano, 2019; Wielockx et al., 2019). The JAK/STAT signaling pathway controls about 50 cytokine signals to orchestrate hematopoiesis (Staerk and Constantinescu, 2012; Morris et al., 2018). MAPK signaling is involved in the generation of hematopoietic stem and progenitor cells (HSPC), erythropoiesis, mylogenesis (Geest and Coffer, 2009). TGF-β signaling can influence the generation of HSCs (Blank and Karlsson, 2015). Some of these genes were not directly relevant to hematopoiesis, which mechanisms need further research to discover. For example, many transcript factors, such as *FOS, EGR1, FOXG1, TF*, and other types of genes, such as MT1 family (Metallothionein), *DDX60* (Probable ATP-Dependent RNA Helicase), *MX1*(Interferon related GTPase), indicated a close couple to the promotion effects caused by culturing hESCs with cSAP. However, the mechanism of cSAP induced biological changes is unclear. The relationship between changing transcriptional profiles and promoting hematopoiesis needs to be elucidated using further analysis such as gene sequence and proteomics at a single cell level.

## 5 Conclusion

The hemopoietic potential of hESCs is critical in blood generation and related regenerative medicine. This study demonstrates that the adhesion and pluripotency of hESCs are crucial in subsequent hemopoietic differentiation. cSAP can be a new family of artificial ECMs (protein- or peptide-modified cSAPs) where the surface presents complex physical and chemical cues. cSAP modulate hESC adhesion and the ability to conduct hemopoietic differentiation. By merely manipulating hESCs using cSAPs, the number of hematopoietic progenitors and erythroid progenitors can be enhanced 3–4 folds. Altogether, cSAP could be the next-generation tool for hESC expansion and blood cell generation.

## Data Availability

The RNAseq data presented in the study are deposited in the Array Express repository, accession number E-MTAB-11123.
