# Supplementary figures and images for: Colloidal Self-Assembled Patterns Maintain the Pluripotency and Promote the Hemopoietic Potential of Human Embryonic Stem Cells
Source: Front Cell Dev Biol. 2021 Nov 16;9:771773. doi: 10.3389/fcell.2021.771773 (PMC8636751; doi:10.3389/fcell.2021.771773)

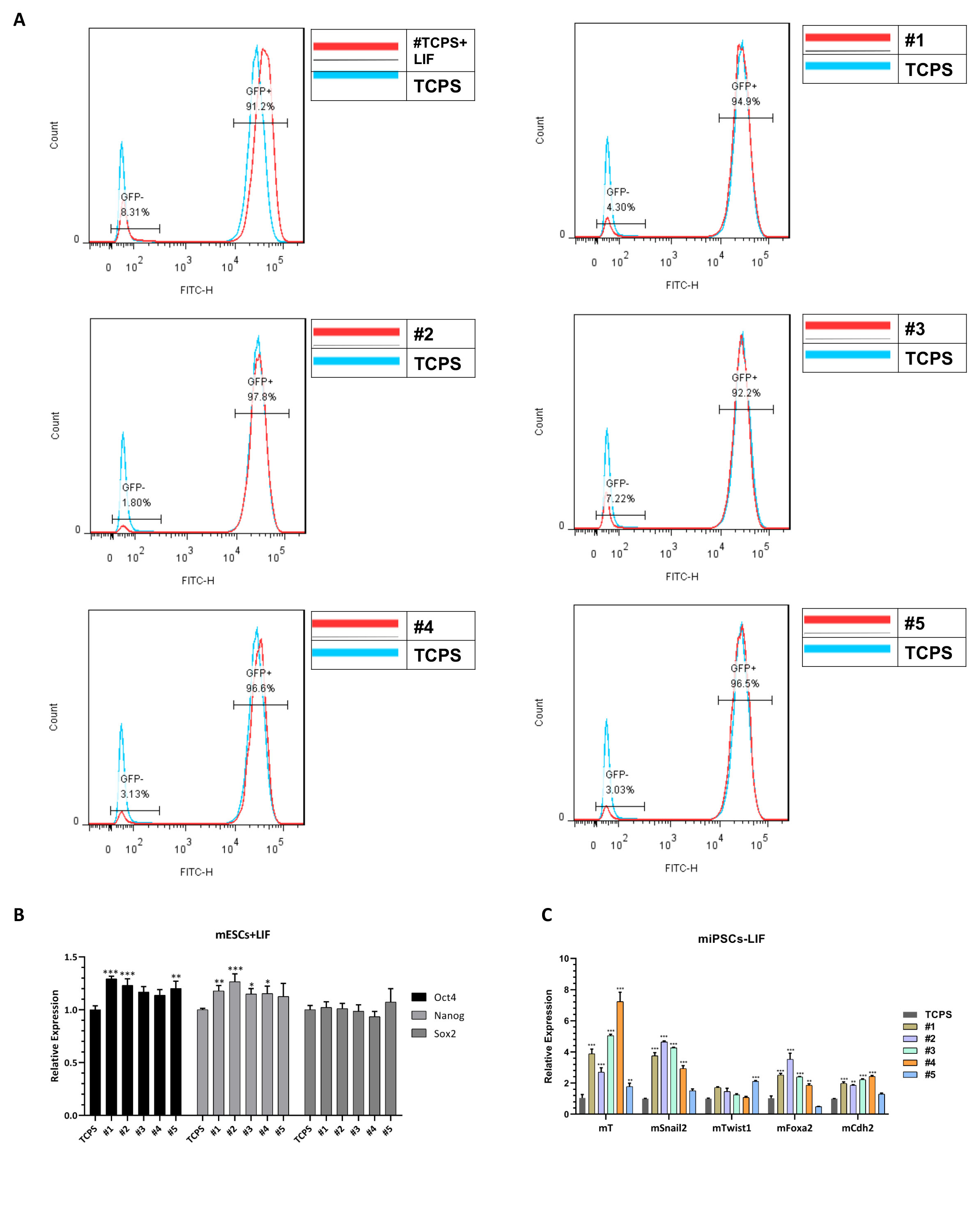

Supplement: Supplementary file 1 [file Image1.JPEG]

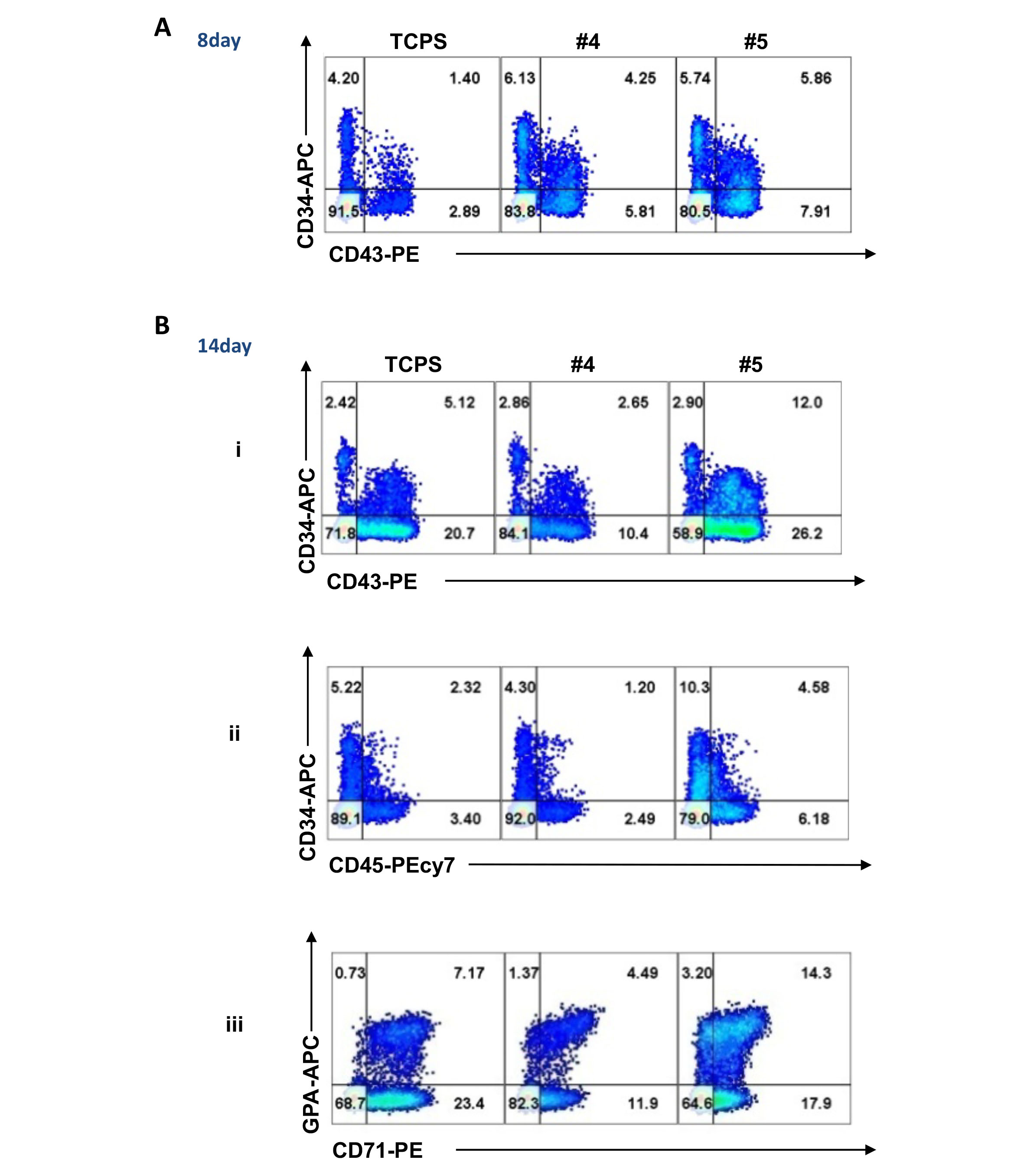

Supplement: Supplementary file 2 [file Image2.JPEG]
