# Supplementary material for: Colloidal Self-Assembled Patterns Maintain the Pluripotency and Promote the Hemopoietic Potential of Human Embryonic Stem Cells
Source: Front Cell Dev Biol. 2021 Nov 16;9:771773. doi: 10.3389/fcell.2021.771773 (PMC8636751; doi:10.3389/fcell.2021.771773)
Supplement: Supplementary file 3 [file DataSheet1.docx]

Supporting Information

**Colloidal self-assembled patterns (cSAPs) maintain the pluripotency and promote the hemopoietic potential of human embryonic stem cells (hESCs)**

Jiao Lin^1, #^, Jiahui Zeng^2, #^, Wencui Sun^2, #^, Kun Liu^1^, Myagmartsend Enkhbat^1^, Danying Yi^2^, Jiaxin Liu^2^, Bo Chen^2,^*, Feng Ma^2,^*, Peter Kingshott^3^, Peng-Yuan Wang^1,3,^*

1. Shenzhen Key Laboratory of Biomimetic Materials and Cellular Immunomodulation, Shenzhen Institute of Advanced Technology, Chinese Academy of Sciences, Shenzhen, Guangdong, 518055, China
2. Stem Cell Center, Institute of Blood Transfusion, [Chinese Academy of Medical Sciences](http://www.iciba.com/chinese_academy_of_medical_sciences) & Peking Union Medical College (CAMS & PUMC), Chengdu, Sichuan, 610052, China
3. Department of Chemistry and Biotechnology, Swinburne University of Technology, Hawthorn, Victoria 3122, Australia

**Figure S1** (A) *Oct4-*GFP^+^ miPSCs cultured on cSAPs (#1/#2/#3/#4/#5) without LIF and flat control (TCPS) with or without LIF for 7 days and used to analyze the percentage of *Oct4*-GFP^+^ cells by FACS; (B) pluripotent markers’ expression in mESCs; (C) mesoderm markers’ expression in miPSCs.

**Figure S2** H1 hESCs were cultured on cSAPs (#4 and #5) or flat control (TCPS) prior to be further co-cultured with AGM-S3 cells,and then subjected to FACS analysis using the following combinations of antibodies: (A) anti-CD34/CD43 (at D8), and (B) anti-CD34/CD43, anti-GPA/CD71, anti-CD34/CD45 (at D14). Most classic hematopoietic populations were improved by pre-culturing on cSAPs #5, which indicated that hematopoietic potentials of H1 hESC were significantly promoted.

**Table S1: Primers used for Real-Time PCR.**

**Table S2: Immunofluorescence staining antibodies and Flow antibodies**

**Figure S1**


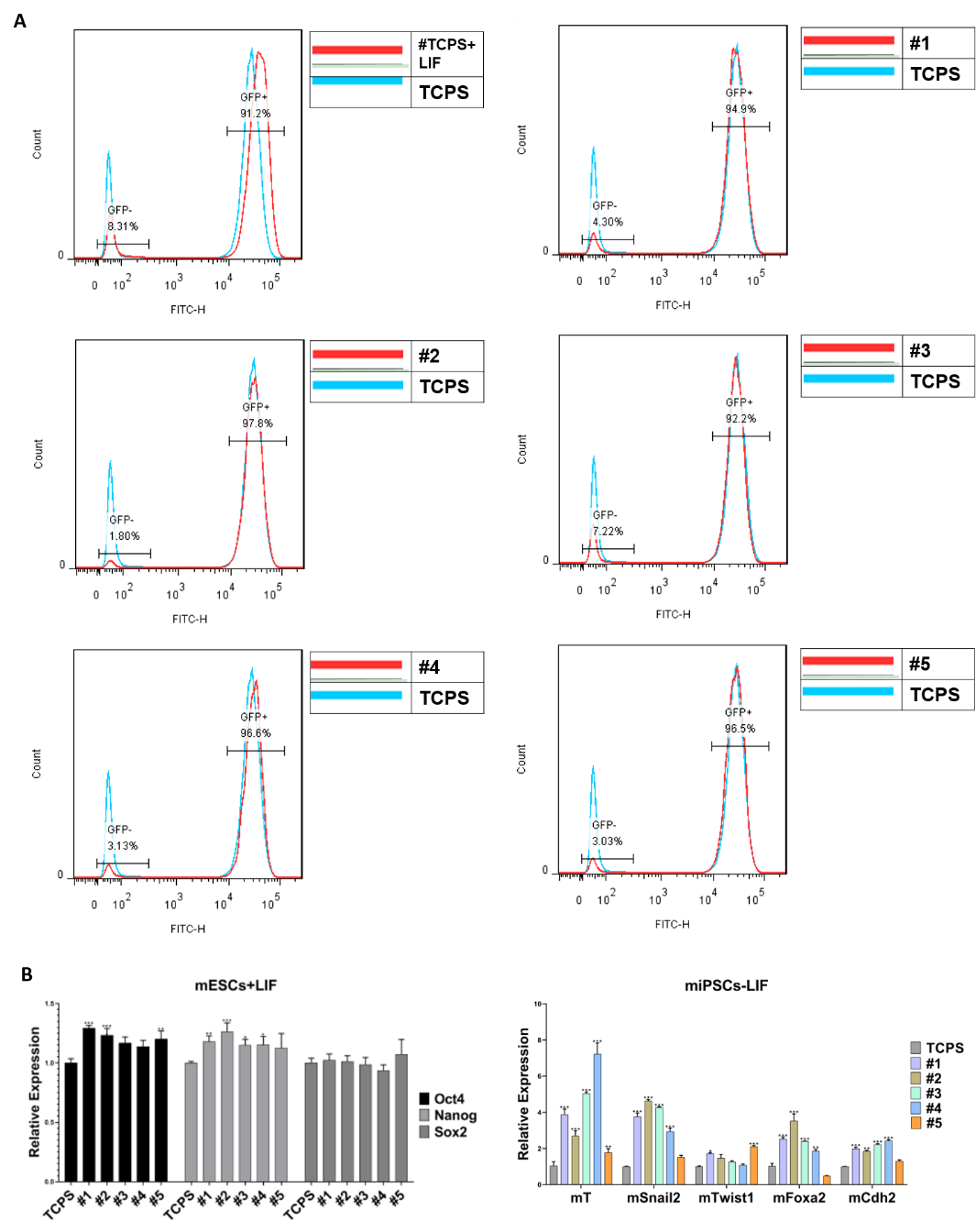


**Figure S2**


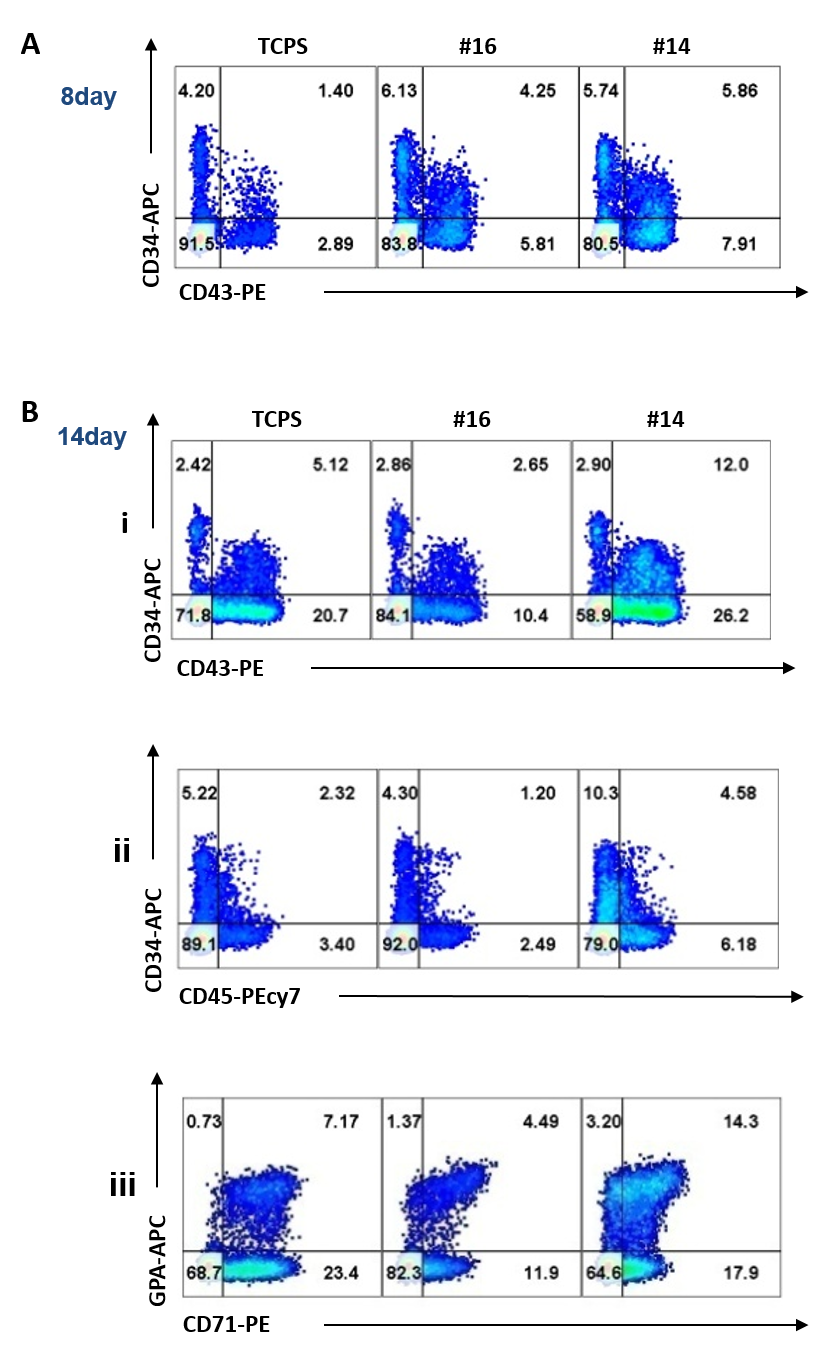


**Table S1: Primers used for Real-Time PCR.**

| Name | Sequence (5’-3’) | Length |
| --- | --- | --- |
| mSox2-F | CCgCgTCAAgAggCCCATgAA | 21bp |
| mSox2-R | CCgCTTCTCggTCTCggACAA | 21bp |
| *mOCT4-*F | TCTTTCCACCAggCCCCCggCTC | 23bp |
| *mOCT4-*R | TgCgggCggACATggggAgATCC | 23bp |
| *mNanog-*F | AgggTCTgCTACTgAgATgCTCTg | 24bp |
| *mNanog-*R | CAACCACTggTTTTTCTgCCACCg | 24bp |
| *mBrachyury(T)-*F | gAACCTCggATTCACATCgT | 20bp |
| *mBrachyury(T)-*R | TTCTTTggCATCAAggAAgg | 20bp |
| *mSnail2-*F | ATCCTCACCTCgggAgCATA | 20bp |
| *mSnail2-*R | TgCCgACgATgTCCATACAg | 20bp |
| *mTwist1-*F | AgCgggTCATggCTAACg | 18bp |
| *mTwist1-*R | ggACCTggTACAggAAgTCgA | 21bp |
| *mFoxa2-*F | CCCTACgCCAACATgAACTCg | 21bp |
| *mFoxa2-*R | gTTCTgCCggTAgAAAgggA | 20bp |
| *mCdh2-*F | ggCgTCTgTggAggCTTCT | 19bp |
| *mCdh2-*R | ggAAATCCAgTCTTgCATAATgC | 23bp |
| *mGapdh-*F | ACCTgCCAAgTATgATgACATCA | 23bp |
| *mGapdh-*R | CCCTCAgATgCCTgCTTCAC | 20bp |

**Table S2: Immunofluorescence staining antibodies and Flow antibodies**

| Antibodies | Brand | Antibody species | Cat |
| --- | --- | --- | --- |
| OCT4 | Santu Cruz | Mouse | Sc-5279 |
| SOX2 | Santu Cruz | Mouse | Sc-356823 |
| SSEA4 | Santu Cruz | Mouse | Sc-59368 |
| FITC-IgG(H+L) | BOSTER | Goat | BA1101 |
| CD34 | BD | APC | 555824 |
| CD43 | BD | PE | 560199 |
| CD45 | BD | PE-CY7 | 557748 |
| CD235a(GPA) | BD | APC | 551336 |
| CD71 | BD | PE | 561938 |
| 7-AAD | BD | 7-AAD | 559925 |
